# A Study on the Relationship Between Adolescent Health Behavior, BMI, and Blood Physical and Chemical Properties

**DOI:** 10.3389/fpubh.2022.766101

**Published:** 2022-03-15

**Authors:** Jie Peng, Lian Yin, Kun Wang, Tingran Zhang, Hengxu Liu, Jinxin Yang, Jiong Luo

**Affiliations:** ^1^School of Physical Education, Liupanshui Normal University, Liupanshui, China; ^2^Research Centre for Exercise Detoxification, College of Physical Education, Southwest University, Chongqing, China

**Keywords:** health behavior, body mass index, blood biochemical indexes, health promotion, adolescents, correlation analysis

## Abstract

In this study, the blood test index, demographic data, and health promotion behavior of adolescents were analyzed to provide a reference for early prevention and treatment of physical decline and abnormal biochemical indexes of adolescents. Using a cross-sectional study design, 1,436 valid samples were obtained by stratified random sampling, and the data were processed by SPSS21.0 statistical analysis software. The results showed that the overall health-promoting lifestyle of adolescents was good, and the interpersonal support behavior was the best, and the health responsibility and sports participation behavior were the worst; the interpersonal support and sports participation behavior of adolescents with normal weight were significantly better than those with overweight or light weight, while the overall health-promoting behavior of adolescents with high fasting blood glucose (FBG) before meals was poor, those with high glutamate pyruvate transaminase (GPT) had poor nutritional behavior and health responsibility behavior, while those with high uric acid (UA) had poor interpersonal support and stress coping behavior. The overweight rate and abnormal detection rate of UA and triglyceride (TG) in boys were significantly higher than those in girls, and the higher BMI of teenagers, the higher abnormal detection rate of GPT, UA, and TG, the better nutritional behavior, health responsibility behavior, and sports participation behavior, the lower abnormal detection rate of GPT, UA, and TG; the higher education level of parents, the better teenagers' sports participation and health responsibility behavior, the lower the incidence of overweight, the more time they spend playing online games and drinking sugary drinks on weekdays (or holidays), the higher the incidence of overweight.

## Introduction

In 1995, the Ministry of Education of China began to respond to the pilot work of the WHO on promoting health-promoting schools. It preferred 12 schools in Chifeng, Wuhan, and Beijing as practice units and began to carry out the comprehensive establishment of health promotion schools from different entry points, such as the prevention of tobacco use, vision decline, obesity prevention, and nutrition (1, 2). After 2014, health promotion schools began to enter the national promotion stage based on the construction of health promotion counties and districts. In October 2016, the Fifth Plenary Session of the 18th CPC Central Committee put forward “Healthy China 2030 Planning Outline”, which put forward “strengthening the integration of sports and medicine and non-medical health intervention and promoting the formation of disease management and health service mode of integration of sports and medicine”. According to the spirit of “program”, schools should bring health education into the national education system, let teenagers take physical exercise as a way of life, and focus on primary and secondary schools to establish a school health education promotion mechanism, so that the majority of teenagers can obtain the maximum health benefits (3–5).

As we all know, with the change of human disease etiology and health intervention mode from “disease treatment” to “disease prevention”, the use of physical exercise for disease treatment and prevention has become a common means and method in the world. The core of the integration of sports and medicine in healthy China (National Health) and sports power (National Fitness) is to build a sports promotion health service system covering the whole population and the whole life cycle. Among them, the promotion of national fitness to health is mainly achieved by guiding people to form scientific fitness behavior and improving scientific fitness level, while the means and methods of national health are mainly achieved through medical intervention, health education, and behavior intervention, and creating a healthy environment. Health promotion behavior is an essential element of a healthy lifestyle. By actively establishing the concept of health promotion of all ages, we can comprehensively promote the positive health behavior of the people (6, 7). Compared with children and adults, adolescents are more suitable to guide healthy behaviors and carry out health promotion activities, to establish their healthy lifestyle (8). Because the root of many serious diseases in adulthood may come from the unhealthy life of teenagers, which may lead to chronic diseases, cancer, or premature death, and relatively increase the medical expenses (9, 10).

According to relevant research reports, in recent years, chronic diseases have gradually become younger, and some teenagers are even in the state of living with diseases (11). Chronic diseases are usually associated with obesity. Obesity in adolescence will increase the risk of obesity and chronic diseases in adulthood (12, 13). Overweight or obese adolescents are more likely to suffer from metabolic syndrome than normal-weight people, with abnormal blood glucose, fatty acid glyceride, cholesterol, and other biochemical abnormalities (14, 15). Therefore, from the perspective of three stages and five levels of epidemiological disease prevention, early and effective prevention of obesity can prevent or delay the occurrence of a variety of chronic diseases at the same time and maintain the physical and mental health of adolescents (16). At present, the lifestyle and obesity of adolescents in China have been considered as the most important factors affecting their health and illness. Therefore, it is the primary task to evaluate the early lifestyle of adolescents, such as diet, exercise, cleanliness, stress, sleep, and whether they have obesity and health problems caused by unhealthy health behaviors, and It is not only the primary task of implementing effective health promotion behavior but also the simplest way for teenagers to invest in health and health management (17). Based on this, to implement the provisions of the health promotion school plan, Chongqing Municipal Education Department has increased the supervision of youth health management and health examination and conducted a comprehensive physical examination every year when the primary and secondary school freshmen enter the school. Through random sampling of the experience data of junior high school freshmen in the winter of 2019, this study obtains the personal background and blood biochemical indicators of the relevant samples. Through analyzing the relationship between the health status, health promotion behavior, and physical indicators of teenagers, it is expected to find out the reasons that affect the health of teenagers, to take more effective measures to help teenagers to develop lifelong health behavior to provide a useful reference for the habit.

## Research Objects and Methods

### Respondents

With the support and help of the school administration and the relevant departments of the Municipal Education Bureau, the research group finally obtained the health examination reports of junior middle schools (freshmen) in Chongqing. By adopting the stratified and class-based random sampling method, 30 classes (all students participated in the blood sampling) were selected by them, and 1,436 respondents were obtained. A questionnaire survey was conducted from September 2018 to October 2018.

### Questionnaire Design

A structured questionnaire was designed, which consisted of four parts, which are as follows:

#### Demographic Data

Including personal and family life, such as age, gender, height, weight, family structure, history of the disease, education level of parents, diet, nature, and frequency of intake of sugary drinks, time of watching TV, surfing the Internet, and playing video games.

#### Adolescent Health Promotion Behavior Scale

It was first proposed by Pender and then modified by Walker in 1995. The Chinese version of the health-promoting lifestyle assessment scale was translated and widely used (18, 19). The original scale has 48 items and six dimensions. After the pre-test, through the division and internal consistency test, eight questions were eliminated. The number of items corresponding to the six dimensions is as follows: interpersonal support (7), stress response (6), nutritional behavior (6), life appreciation (8), sports participation (5), and health responsibility (8). Using the Likert five-grade scoring method, the higher the score, the higher the level of health promotion behavior. The results of the pre-test showed that the intrinsic consistency reliability of the total subscale was 0.81, and the α values of six dimensions were 0.85, 0.83, 0.78, 0.80, 0.88, and 0.76, respectively. The corresponding Cronbach's α values from the official investigation and recovery data were also distributed between 0.77 and 0.86. It shows that the reliability of the test results is good.

#### Biochemical Index

The indicators were obtained from the physical examination reports of the students (blood sampling results and diagnosis report provided by the hospital after blood sampling examination). The blood biochemical indexes included fasting blood glucose (FBG, reference value 65–120 mg/dl), triglyceride (TG, reference value 35–165 mg/dl), total cholesterol (TC,reference value 120–200 mg/dl), creatinine (CRE, reference value 0.4–1.4 mg/dl), uric acid (UA, reference value 2.5–7.6 mg/dl), glutamate pyruvate transaminase (GPT,reference value 0–40 U/L).

#### Body Structure Measurement

Body mass index (BMI) was calculated. According to the definition of obesity in children and adolescents in China, the subjects were divided into four grades: light, normal, overweight, and obese.

### Mathematical Statistics

SPSS 17.0 was used to establish and analyze the database, and the appropriate statistical methods were selected according to the research purpose. There were descriptive analysis, one-way ANOVA, Mann-Whitney U test, Kruskal-Wallis test, correlation analysis, and multiple logistic regression analysis. The significant level of all indexes was set as α = 0.05.

## Results

### Analysis of the Influence of BMI on Health Promotion Behavior of Adolescents

Table 1 shows:

(1) The average score of each item of the six dimensions of health promotion behavior is between 3.09 and 3.68. As for the overall health promotion behavior, the average score of each item is 3.44, which is higher than the median value of each item of 3, which belongs to the upper-middle level, indicating that the subjects' health promotion lifestyle belongs to the good condition.
(2) From the average score index of each dimension, the “interpersonal support” score was high(73.51%), the “health responsibility” score was the lowest (62.11%), followed by sports participation(63.54%).
(3) From the relationship between health promotion behavior and BMI, the total average score of six dimensions of health promotion behavior in the normal-weight group (138.97 ± 23.05) was significantly higher than that in the underweight group (135.51 ± 24.47), and that in the underweight group was significantly higher than that in the overweight group (131.19 ± 25.47). Furthermore, from the perspective of six dimensions, the performance differences of health promotion behavior only exist in social interpersonal support and sports participation behavior, and both show that the normal weight is better than the underweight, and the underweight is better than the overweight.

**Table 1:** The influence of body mass index on health promotion behavior of adolescents.

**Variable name**	**The average score of each question**	**Mean value of score index%**	**Underweight (A)**	**Normal weight** **(B)**	**Overweight and obesity (C)**	**Test** **(F)**	**LSD multiple comparisons**
Interpersonal support	3.68 ± 0.91	73.51 ± 12.15	25.44 ± 4.89	26.15 ± 6.74	24.12 ± 5.12	6.78**	b>a>c
Stress response	3.55 ± 0.84	72.74 ± 15.12	21.51 ± 4.06	21.95 ± 4.72	21.32 ± 4.74	1.54	a=b=c
Nutritional behavior	3.51 ± 0.81	70.89 ± 11.87	21.01 ± 3.91	21.46 ± 3.77	21.21 ± 4.85	0.81	a=b=c
Life appreciation	3.57 ± 0.89	70.17 ± 14.65	28.22 ± 8.15	28.77 ± 7.05	27.56 ± 7.21	1.58	a=b=c
Sports participation	3.25 ± 0.83	63.54 ± 14.69	15.54 ± 4.29	16.77 ± 4.15	14.84 ± 5.21	5.09**	b>a>c
Health responsibility	3.09 ± 0.86	62.11 ± 13.17	24.31 ± 6.09	24.81 ± 5.58	24.45 ± 5.10	1.67	a=b=c
Total health promotion score	3.44 ± 0.89	68.82 ± 12.53	135.51 ± 24.47	138.97 ± 23.05	131.19 ± 25.47	4.88**	a>b>c

calculation method of score index = (average score of all levels ÷ Full Score) × 100%; The “*” and “**” indicates the significant level of 0.05 and 0.01 respectively; in multiple comparison of LSD, A = B =C means that there is no difference in certain health promotion behaviors among the three groups. On the contrary, if A > B >C, it means that a certain behavior of group A is better than group B, and group B is better than group C.

### Analysis of the Relationship Between Blood Biochemical Indexes and Health Promotion Behavior in Adolescents

Table 2 shows:

(1) According to the classification of TG and TC, social interpersonal support, stress coping, nutritional behavior, life appreciation, sports participation, health responsibility, and overall health promotion behavior did not affect TG and TC.
(2) Fasting blood glucose before meals seems to be the most affected by adolescent health promotion behavior. The higher FBG, the lower social interpersonal support (*X*^2^ = 2.15, *p* < 0.05), the lower stress coping ability (*X*^2^ = 3.27, *p* < 0.05), the worse nutritional behavior (*X*^2^ = 2.94, *p* < 0.05), the worse life appreciation ability (*X*^2^ = 2.56, *p* < 0.05), and the worse sports participation behavior (*X*^2^ = 3.19, *p* < 0.05), The worse the health responsibility behavior (*X*^2^ = 2.65, *p* < 0.05), the worse the overall health promotion behavior (*X*^2^ = 3.57, *p* < 0.05).
(3) Glutamate pyruvate transaminase was only affected by the two dimensions of nutritional behavior and health responsibility behavior. The higher the GPT, the worse the nutritional behavior (z = −2.16, *p* < 0.05) and health responsibility behavior (z = −2.43, *p* < 0.05).
(4) Uric acid was only affected by social interpersonal support and stress-coping behavior. The higher UA, the better social interpersonal support behavior (z = 5.77, *p* < 0.01), and the worse stress-coping behavior (z = 1.98, *p* < 0.05).

**Table 2:** Analysis of the relationship between the five major blood biochemical indexes and health promotion behavior in adolescents.

		**Interpersonal support**		**Stress response**		**Nutritional behavior**		**Life appreciation**		**Sports participation**		**Health responsibility**		**Total score of promotion**	
		**Mean value**	**X^**2**^/z**	**Mean value**	**X^**2**^/z**	**Mean value**	**X^**2**^/z**	**Mean value**	**X^**2**^/z**	**Mean value**	**X^**2**^/z**	**Mean value**	**X^**2**^/z**	**Mean value**	**X^**2**^/z**
^#^GPT	Normal	21.29	−0.12	25.65	−1.12	24.87	–2.16*	27.89	−1.36	15.89	−0.76	22.39	–2.43*	137.03	−1.83
	Overtop	21.45		24.39		22.33		26.86		15.01		19.91		128.44	
^&^FBG	Too low	21.31	2.15*	24.89	3.27*	24.32	2.94*	27.78	2.56*	15.74	3.19*	21.32	2.56*	134.15	3.57*
	Normal	21.65		25.47		24.67		28.36		16.54		21.77		135.95	
	Overtop	16.47		13.66		12.51		20.25		8.85		14.63		91.77	
^&^UA	Too low	18.16	5.77**	28.19	1.98*	21.12	1.73	31.69	0.69	15.09	1.59	18.61	1.35	129.06	0.36
	Normal	22.65		25.77		23.17		29.77		15.61		21.32		135.85	
	Overtop	23.17		23.15		24.49		28.85		14.92		22.11		136.05	
^&^TG	Too low	21.58	0.38	25.00	0.51	25.56	0.57	28.29	0.15	17.62	1.56	21.74	0.90	138.25	0.59
	Normal	21.21		25.26		24.39		28.31		16.21		21.43		137.65	
	Overtop	22.06		26.23		24.56		28.99		15.87		22.24		149.77	
^&^TC	Too low	21.19	0.71	24.87	0.78	24.46	0.39	27.09	1.52	15.79	0.05	20.91	0.89	133.95	1.33
	Normal	21.55		25.39		24.33		28.88		15.73		21.60		126.56	
	Overtop	20.60		24.61		24.81		27.91		15.59		22.01		134.74	

For blood indexes GPT, FBG, UA, TG, TC, “#” means using two independent samples of non-parametric test, Mann-Whitney test, Z statistics; “&” multiple independent samples of non-parametric test, Kruskal-Wallis test, chi square value; *p < 0.05, **p < 0.01, ** p < 0.001.

### Analysis of the Relationship Between Daily Living Habits, Leisure Time Arrangement, and Health Promotion Behavior of Adolescents

Table 3 shows:

(1) In terms of living habits, the time spent in watching TV or playing online games was negatively correlated with the six dimensions of health promotion behavior and the total score of health promotion behavior in most cases. It was also significantly correlated with BMI (positive or negative), which seems that the longer the time of watching TV and playing games, the lower the scores of nutritional behavior (*r* distribution range −0.17^**^~-0.25^**^), social interpersonal support (*r* distribution range −0.11^**^~ −0.17^**^), health responsibility behavior (*r* distribution range −0.15^**^~ −0.24^**^), and life appreciation behavior (*r* distribution range −0.15^**^~ −0.24^**^) and the lower the scores of exercise behavior (*r* distribution range −0.13^**^~ −0.21^**^), stress coping behavior (*r* distribution range −0.10^**^~ −0.15^**^), and overall health promotion behavior (*r* distribution range −0.13^**^~ −0.24^**^) were.
(2) The more times adolescents eat breakfast or dinner with their family each week, the more positively correlated with the 6-dimension score of health promotion behavior and the overall score of health promotion behavior (*r* distribution interval: 0.09^*^ to 0.29^**^), but also negatively correlated with BMI (*r* distribution interval: −0.11^*^ to −0.15^**^).
(3) The more times and quantity of sugary drinks the adolescents drink per week, the more negative correlation with the 6-dimension score of health promotion behavior and the overall score of health promotion behavior(r distribution interval: −0.10^*^ to −0.28^**^), but which has a significant positive correlation with BMI (*r* = 0.15^**^).

**Table 3:** Statistical table of correlation matrix between daily living habits, leisure time arrangement, and health promotion behavior of adolescents.

	**Interpersonal support**	**Stress response**	**Nutritional behavior**	**Life appreciation**	**Sports behavior**	**Health responsibility**	**Overall promotion**	**BMI**
Daily TV time (h)	−0.17**	−0.15**	−0.25**	−0.13**	−0.21**	−0.20***	−0.24***	0.06
Holiday TV time (h)	−0.11**	−0.06	−0.17**	−0.06	−0.16**	−0.16***	−0.13***	0.08*
weekdays online games time (h)	−0.14**	−0.12**	−0.21**	−0.10*	−0.13**	−0.15***	−0.18***	0.08*
Holiday online game time (h)	−0.16**	−0.10*	−0.23**	−0.11**	−0.15**	−0.24***	−0.17***	0.14***
Breakfast with family every week (Times)	0.13**	0.12**	0.29**	0.10*	0.13**	0.22***	0.19***	−0.15**
Weekly dinner with family (Times)	0.11**	0.10**	0.21**	0.14**	0.09*	0.15**	0.15***	−0.11**
Drink sugary drinks every day (Times)	−0.16**	−0.14**	−0.28**	−0.15**	−0.13**	−0.22***	−0.21***	−0.04
Drink sugary drinks every time(cups)	−0.12*	−0.05	−0.10**	−0.02	−0.04	−0.16**	−0.13*	0.15**

*p < 0.05, ** p < 0.01, *** p < 0.001.

### Predictive Analysis of Background Factors, Living Habits, and Health-Promoting Behaviors on BMI and Biochemical Factors in Adolescents

Many factors are influencing the BMI and blood biochemical indexes of adolescents, such as genetic factors, family factors, personal habits, extracurricular leisure time allocation, and health promotion behavior. If we want to find out the key factors from these factors, logistic multiple regression analysis is the best way. Because the application premise of the logistic method is that the dependent variable type is classified variable, and the influencing variable (independent variable) can be either continuous variable or classified variable, and the requirements of influencing factors do not have to meet the independent, normal, and other conditional assumptions.

According to the distribution characteristics of biochemical indexes, the biochemical indexes of subjects can be classified as “abnormal” or “normal”, and BMI can be classified as “overweight” or “non-overweight”, so all the predictive variables become typical binary variables. Due to the number of subjects with abnormal FBG and TC being less than normal, they were not included in the regression analysis. It is assumed that the dependent variable y follows a binomial distribution, and the value of the binomial distribution is (1,0), y = 1 (that is abnormal). In this study, 18 independent variables, such as gender, parents' education level, and family structure, are selected, and the forced introduction method is used for two classifications logistic regression, and four regression equations are obtained. The results are shown in Table 4, from which it is not difficult to find:

(1) In the BMI regression model, only seven of the 17 variables in the regression equation reached a significant level. From the odds ratio (OR), it is not difficult to find that the risk of being overweight for boys is 2.034^**^ times higher than that for girls; the risk of being overweight for their children will increase to 1.859^**^ times of the original level when their parents' education level is reduced by one level; the risk of overweight will decrease to 0.631^**^ times and 0.893^**^ times of the original level, respectively, when their scores of sports participation behavior and health responsibility behavior increase by one point. If the time of playing online games on weekdays and holidays increased by 1 h and the time of drinking sugary drinks increased by one time, the probability of overweight was increased to 1.233^**^, 1.206^**^, and 1.264^**^ times of the original level, respectively.
(2) Among the three predictive models of biochemical indexes, only four of the 18 variables in the GPT model reached a significant level. Among them, the risk of abnormal GPT will increase 1.232^**^ times following BMI of teenagers increases one unit; the risk of abnormal GPT will decrease 0.881^**^, 0.886^**^, 0.866^**^ times respectively with the increase of one unit of nutrition behavior, sports participation behavior, and health responsibility behavior.
(3) Five of the 18 variables in the UA regression model reached the significant level, in which gender had the most significant effect, which showed that the risk of abnormal UA in boys was 4.293^**^ times higher than that in women when BMI was increased by one unit, the risk of abnormal UA was increased by 1.214^**^ times. while the scores of nutritional behavior, exercise behavior, and health responsibility behavior were increased by one point, the probability of abnormal UA in adolescents was decreased to 0.893^**^, 0.900^**^, and 0.882^**^ times of the original level, respectively.
(4) Eight of the 18 variables in the total TG regression model reached a significant level, which showed that the risk of abnormal TG in men was 1.923^**^ times higher than that in women. If the BMI of adolescents increases by one unit, the time of playing online games increases by 1 h, the number of sugary drinks increases by one time every day and the number of sugary drinks increases by one cup every time, the risk of abnormal TG will be increased to 1.198^**^, 1.126^**^, 1.240^**^, and 1.166^**^ times of the original level respectively. While the score of nutrition behavior and sports behavior was increased by one point, the probability of abnormal TG was decreased to 0.815^**^ and 0.837^**^ times of the original level, respectively.

**Table 4:** Statistical table of multiple regression model of adolescent background factors, living habits, and health promotion behavior on BMI and biochemical factors.

	**Regression model 1 (BMI) Overweight/non overweight**		**Regression model 2 (GPT)** **Abnormal/normal**		**Regression model 3 (UA) Abnormal/normal**		**Regression model 4 (TG)** **Abnormal/normal**	
	**β**	**OR**	**β**	**OR**	**β**	**OR**	**β**	**OR**
Constant	−1.47		−7.50		−0.86		−2.48	
Gender	0.710	2.034**	0.007	1.007	1.457	4.293**	0.654	1.923**
Parents' education level	0.620	1.859**	0.013	1.013	0.012	1.012	−0.701	0.496**
Family structure	0.040	1.041	0.011	1.011	0.008	1.008	−0.018	0.982
Body mass index (BMI)			0.209	1.232**	0.194	1.214**	0.181	1.198**
Interpersonal support	−0.040	0.961	−0.003	0.997	0.034	1.035	−0.022	0.978
Stress response	−0.050	0.951	−0.007	0.993	−0.065	0.937	−0.036	0.965
Nutritional behavior	−0.073	0.930	−0.127	0.881**	−0.113	0.893**	−0.205	0.815**
Life appreciation	−0.034	0.967	−0.013	0.987	−0.004	0.996	−0.047	0.954
Sports behavior	−0.461	0.631**	−0.121	0.886**	−0.105	0.900**	−0.178	0.837**
Health responsibility	−0.113	0.893**	−0.144	0.866**	−0.126	0.882**	−0.052	0.949
Daily TV time (h)	0.030	1.030	0.003	1.003	0.081	1.084	0.061	1.063
Holiday TV time (h)	0.040	1.041	0.010	1.010	0.039	1.040	0.033	1.034
Weekdays online game time (h)	0.201	1.223**	0.007	1.007	0.057	1.059	0.119	1.126**
Holiday online game time (h)	0.187	1.206**	0.001	1.001	0.041	1.042	0.026	1.026
Breakfast with family every week (Times)	0.014	1.014	−0.002	0.998	0.002	1.002	0.001	1.001
Weekly dinner with family (Times)	0.022	1.022	−0.003	0.997	0.006	1.006	−0.008	0.992
Drink sugary drinks every day (Times)	0.234	1.264**	−0.043	0.958	0.030	1.030	0.215	1.240**
Drink sugary drinks every time (cups)	0.007	1.007	−0.032	0.969	0.071	1.074	0.154	1.166**

*p < 0.05, **p < 0.01.

## Discussion

### On the Factors Influencing BMI of Teenagers

This study shows that the obesity rate of male students is higher than that of female students, and the risk of being overweight is 1.93 times higher than that of female students. This result is similar to the research of many scholars at home and abroad (20–23), which means that in the current school health promotion plan in China, personalized promotion strategies should be developed for high-risk cases or gender differences. This study also found that when the parents' education level is reduced by one level, the risk of overweight of their children will be increased to 1.859 times of the original. According to the research of Gali et al. (24), it is also found that the overweight rate of children of parents with an education level below junior college is significantly higher than that of parents with a bachelor's degree or above. In addition, KuoLiong et al. (25) reported that the higher the education level, the better the score of nutrition knowledge. There are also inconsistent reports on the effect of parental education on overweight adolescents. Martin et al. found that (26) those whose mothers' education level is above junior college have a higher probability of abnormal TG of their children than those with low education level. The scholar explains that mothers with higher education pay more attention to their children's nutritional supplements and are more likely to eat delicate, high calorie, and high-fat meals, thus, increasing the incidence of abnormal biochemical indicators. It seems that more research is needed to confirm whether parents' education level has a positive or negative impact on their children's overweight or TG.

A large number of previous studies support the existence of a correlation between physical activity and obesity. Physical activity can indeed reduce body fat, maintain or avoid the loss of lean weight, and improve cardiopulmonary endurance (27, 28); and enhancing health responsibility behavior can increase the ability of individual weight control (29) and can effectively control the incidence of obesity in adolescence (30). This study found that every one-point increase in the scores of sports participation behavior and health responsibility behavior would reduce the probability of overweight to 0.631 times and 0.893 times of the original level, which further confirmed the findings of the above scholars. Regression model 1 also showed that whether it was weekdays or holidays, the risk of overweight was increased to 1.233, 1.206, and 1.264 times of the original level when the time of playing online games and drinking sugary drinks increased by 1 h every day. Similar research reports, such as trainer SS (31), found that the risk of overweight among adolescents who play games every day is 1.35 times that of adolescents who do not play games; Chen et al. (32) also found that overweight adolescents spend more time in watching TV and playing online games than non-overweight adolescents, and overweight adolescents perform less health promotion behaviors than non-overweight adolescents. In a word, teenagers' excessive static life and poor performance of health promotion behaviors are all related to their weight; while teenagers' overweight, the lower the implementation rate of health promotion behaviors, the more serious the static lifestyle, the more time they watch TV, play online games, and drink sugary drinks.

### On the Factors Influencing the Blood Biochemical Abnormality of Teenagers

This study found that the common factors affecting the abnormal GPT, UA, and TG of adolescents are BMI, nutritional behavior, and exercise behavior. If BMI increases by one unit value, the probability of abnormal GPT, UA, and TG will increase by 1.232, 1.214, and 1.198 times the original level, respectively. When nutritional behavior and exercise participation behavior increase by one point, the probability of abnormal GPT, UA, and TG will decrease to 0.881 and 0.893, 815 times and 0.886, 0.900, 0.837 times of the original level, respectively. According to previous literature reports, da Fonseca et al. (33) pointed out that about 25% of obese children have steatohepatitis, accompanied by abnormal liver function, liver fibrosis, and liver cirrhosis, and these children tend to live a static life with poor nutritional behavior. Kumar and Kelly (34) found that the values of cholesterol, TG, UA, insulin, and leptin in the blood of the obese group were significantly higher than those of the normal-weight group. Pantalone et al. (35) also found that obese children had poor sports participation behavior, and the blood low-density lipoprotein (LDL) cholesterol, UA, and GPT were higher than those of normal-weight children. Therefore, the findings of this study are similar to the previous research results.

In addition, the study also found that there was a significant gender difference in the incidence of abnormal UA and TG, and the risk of abnormal UA and TG in boys was 4.293 and 1.923 times higher than that in women, respectively. In particular, health responsibility behavior has a significant positive effect on GPT and UA. When the score increases by one point, the risk of abnormal GPT and UA will be reduced to 0.866 and 0.882 times, respectively; and the risk of abnormal TG is also affected by teenagers playing online games, drinking sugary drinks every day, and the number of sugary drinks every time

Among them, health responsibility behavior has a significant positive effect on GPT and UA. When the score increases by one point, the probability of abnormal risk of GPT and UA will be reduced to 0.866 and 0.882 times of the original level, respectively; and the risk of abnormal TG is also affected by teenagers playing online games, drinking sugary drinks every day, and the number of sugary drinks every time. However, studies on these aspects lack the support of more previous studies. From a small number of similar reports, if teenagers can improve their physical activity in sports, it can not only promote their health and physical fitness but also prevent osteoporosis, reduce blood pressure, improve blood composition, prevent and treat chronic diseases, promote psychological and social adaptation and many other benefits (22, 25, 36, 37). Because the biochemical indicators in this study are from the hospital physical examination data and limited by the sample size, the research results may underestimate or overestimate the OR of abnormal biochemical indicators in adolescents. In addition, there are not many kinds of literature at home and abroad that use this research perspective to explore, and the comparison of related results is not sufficient, and some aspects need more research to further explore.

## Conclusion

(1) The heavier the weight of teenagers, the higher the proportion of biochemical abnormalities in their blood. The better the healthy behavior of teenagers, the better the biochemical indexes, and the lower the proportion of overweight.
(2) The static lifestyle and health-promoting behavior of adolescents are closely related to their weight, which showed that the heavier the weight, the worse the performance of health promotion behavior; the more prominent the static lifestyle, the more serious the performance of watching TV, playing online games, drinking sugary drinks.

## Data Availability Statement

The datasets presented in this study can be found in online repositories. The names of the repository/repositories and accession number(s) can be found in the article/supplementary material.

## Ethics Statement

This study was reviewed and approved by the Ethics Review Committee of Southwest University. However, this study does not involve human and animal experiments, and written informed consent is not required.

## Author Contributions

JP is mainly responsible for the design of the paper and the preparation of the questionnaire and participates in the writing of the paper. LY, KW, TZ, HL, and JY are mainly engaged in the distribution of the questionnaire and data processing and analysis. JL provided decision-making and financial support for this study. All authors contributed to the article and approved the submitted version.

## Funding

This study was supported by the humanities and social sciences of the Ministry of Education (Grant No: 20YJA890018).

## Conflict of Interest

The authors declare that the research was conducted in the absence of any commercial or financial relationships that could be construed as a potential conflict of interest.

## Publisher's Note

All claims expressed in this article are solely those of the authors and do not necessarily represent those of their affiliated organizations, or those of the publisher, the editors and the reviewers. Any product that may be evaluated in this article, or claim that may be made by its manufacturer, is not guaranteed or endorsed by the publisher.
